# Physical Activity Levels and Barriers Among Young People with Mental Disorders: A Mixed Methods Analysis Supporting the Development of a National Sport Mental Health Clinic †

**DOI:** 10.3390/sports13110399

**Published:** 2025-11-06

**Authors:** Daniel Vella Fondacaro, Paul Mansell, Michela Agius, Karl Apap Gatt, Nicole Borg, Roberto Galea, Catherine Gatt, Gertrude Fenech, Adrian Richard, Caroline Vassallo, Matthew Slater

**Affiliations:** 1 Department of Sport and Sciences, University of Staffordshire, Stoke-on-Trent ST4 2DF, UK; 2 Department of Psychiatry, University of Malta, MSD 2080 Msida, Malta; 3 Mount Carmel Hospital, ATD 9033 Attard, Malta; 4 Mater Dei Hospital, MSD 2090 Msida, Malta

**Keywords:** sport, physical activity, mental health, barriers, mixed methods

## Abstract

Background: While the positive relationship between mental health and physical activity (PA) is well established, numerous barriers are reported. This study analyzed PA levels and associated barriers in young people attending a national child and adolescent mental health service using a quantitatively driven mixed methods design. Methods: From contacted patient families (*n* = 1284) meeting inclusion criteria, 23.67% (*n* = 304; age 12 to 18 years) completed a questionnaire (quantitative component/supplementary qualitative component). Statistical tests and thematic analysis were used to interpret data. Results: 57.24% (*n* = 174) of participants practiced PA/sport. Those in a sporting discipline did more PA overall, and males were almost twice as likely (OR = 1.98) to do PA/sports than females. PA levels were significantly different across mental disorder groups (highest in personality disorders and related traits, and lowest in disruptive behavioral or dissocial disorders). Participants supported the positive association between mental health and athletic performance, including the use of exercise prescriptions. Barriers to PA included excessive screentime, reduced mental health support/awareness, lack of appropriate facilities, financial difficulties, etc. Conclusion: Further research is needed. However, such results will serve to inform the development of the first documented sport mental health clinic for young people.

## 1. Introduction

The World Health Organization (WHO) published new physical activity (PA) and sedentary behavior guidance in 2020, recommending children and adolescents (5–18 years) should carry out a minimum of 60 min of moderate-to-vigorous intensity PA daily [1]. Unfortunately, recent global estimates reported that 27.5% of adults [2] and 81% of adolescents [3] were not meeting WHO PA recommendations [4]. Although numerous studies have documented the positive relationship between PA and mental health [5], a meta-analysis [6] highlighted several barriers that hinder individuals from participating in PA, including low mood, stress, and insufficient social support, while others [7] identified barriers such as lack of company, lack of time, school obligations, and lack of interest. Another systematic review of qualitative studies [8] reported individual factors (such as lack of motivation, physical factors, and lack of understanding), social/relational factors (such as family and friends), PA nature factors (such as fun, school-based PA), life factors, and environmental factors (such as access to PA facilities) as potential barriers or facilitators. Therefore, patients with mental disorders (MDs) may face greater challenges in establishing and maintaining PA routines compared to the general population.

Interpreting data using core sociodemographic constructs is a crucial component of service development and optimization of healthcare in young people (YP) [9]. A systematic review [10] reported lower PA levels in females compared to males, decreasing PA in adolescents with age, lower PA levels in adolescents with lower literacy, and lower PA levels in those making excessive use of screen time, among others. A three-fold increased risk of lower PA levels has been reported in YP diagnosed with a MD compared to the general population, with mood disorder and autism spectrum disorder (ASD) being the most inactive groups [11].

PA has been recognized as a cost-effective therapeutic option for MDs using exercise prescriptions [12]. However, there is a lack of knowledge among clinicians about PA guidelines, effectiveness of exercise programs, and how to promote PA [13]. In 2018, the WHO underscored the need to embed PA education in medical school and healthcare practitioner curricula to help the future healthcare workforce reduce sedentary times in people at risk and/or living with non-communicable diseases [14]. Global and continental initiatives, such as the VANGUARD project in Europe [15], have also been developed to implement PA in these curricula. The incorporation of PA as a therapeutic framework in patient care further complements Engel’s biopsychosocial approach [16] which emphasizes the importance of a holistic mode of practice.

This study was carried out as part of a wider research project developing a national sport mental health service for YP. There have only been a few reported sport mental health service models, such as within the Canadian system [17] and the Swedish system [18]. However, these focused mostly on the adult elite athlete population. Specialist sport mental health clinics are reported to be more effective than the general national health service as they would consist of highly trained professionals reducing fears of athlete stigmatization [19] and team deselection [20]. Furthermore, specialized clinics provide a timelier service when compared to the long waiting lists within general services [21]. Such a service can serve a dual purpose by providing specialized mental health support for athletes while also acting as an advisory resource to encourage and prescribe PA for young mental health patients.

Malta is a Southern European (three-island archipelago) country in the Mediterranean Sea of approximately 316 square kilometers [22]. Comparable to the British National Health Service, the Malta public healthcare service consists of a comprehensive tax-based service comprising a national mental health service, including a child and adolescent mental health (CAMH) department. The primary aim of this study was to compare the PA levels between sociodemographic groups of patients attending a national CAMH service in Malta. Null hypotheses postulated that there were no significant differences between PA and sport status (H_0_^1^), gender (H_0_^2^), age (H_0_^3^), residential locality district (H_0_^4^), educational level (H_0_^5^), and disorder group (H_0_^6^). The lack of support associated with low sociodemographic status and MDs have been reported to be among the most prevalent barriers towards PA [6]. Therefore, the secondary objective was to explore the perceived barriers that prevent young mental health patients from engaging in PA, including the potential connection between barriers to PA and sociodemographic status. The results generated from this study will serve to inform the development of a national sport mental health service by providing an indication on where to prioritize efforts within an already overstretched healthcare system [23].

## 2. Materials and Methods

### 2.1. Participants

Participants were recruited from the Child and Young People Services (CYPS), which is the Malta national CAMH community service. The inclusion criteria for this study were as follows: (1) active cases at the CYPS; and (2) YP of ages 12 to 18 years. This age group was selected based on work by Wylleman and Lavallee [24], who highlighted this approximate age range as the developmental athletic level and the adolescent psychological level in their developmental model of transitions faced by athletes. The parents or caregivers were instructed to complete the questionnaire together with their child. Therefore, for the purpose of this research, the term “participant” refers to the YP including their parents and caregivers as one unit.

### 2.2. Data Collection

A questionnaire was developed for the purpose of this study, including a core quantitative component supported by qualitative data. The quantitative component included demographic data (age, gender, locality), MD, athletic status (whether participant practiced sport), and the Godin Leisure-Time Exercise Questionnaire (GLTEQ) [25] which is a cost-effective, validated measure of PA levels adding together strenuous (multiplying times per week by nine), moderate (multiplying times per week by five), and mild levels of exercise (multiplying times per week by three). The GLTEQ has been reported to have good test–retest reliability with most variables having reliability coefficients of >0.7 and concurrent validity [25]. Supplementary qualitative data were gathered by means of open-ended questions, including the following: one question for the type of sport (“Does the young person carry out any sports? If yes, what?”), two questions for the relationship between PA/sports and mental health (“Do you feel that mental health has a role in sports? How?”, “Do you feel that sports and exercise help with the young person’s mental health? How?”), one question for the barriers to carrying out PA/sports (“What might be stopping the young person from doing more sports and exercise in this country?”), and one question about the potential benefits of a national sport mental health clinic (“Do you feel that a sport and exercise psychology clinic would be useful within the national health service? Why?”). Given that the participants had little knowledge about the conception of such a clinic, the last question was added to give the researchers an inductive indication of what participants perceived by the term “sport mental health clinic”. The authors were aware that barriers to participation in PA/sports were likely to differ between those doing PA and those who did not. Therefore, the answers to the open-ended questionnaires were compulsory to complete the questionnaire; therefore, the whole sample contributed to the qualitative analysis providing a more representative qualitative section.

The questionnaire was available in both the English and the Maltese language as both languages are Malta’s official languages [26]. The questionnaire was translated from the English to the Maltese language, and then reverse translated to English, with the support of a University of Malta language graduate. Readability testing (Flesch Reading Ease = 71.00, Flesch–Kincaid Grade Level = 5.28, The Smog Index = 6.32) indicated that the questionnaire was easily readable for the selected participant age range. The research team conducting the study were medical doctors from the Malta Mental Health Services and supervising sport psychologists from the University of Staffordshire.

### 2.3. Study Design

This study employed a quantitatively driven (QUAN + *qual*) convergent mixed methods design utilizing quantitative data as the core component, supported by qualitative data from open-ended questions [27]. Therefore, from an epistemological standpoint, this study embraced the principles of pragmatism (integrating postpositivist and interpretivist frameworks), allowing the researchers to diverge from a priori reasoning and fixed absolutes, instead focusing on the real facts as they exist and as they pertain to the research question [28].

**Figure 1:**
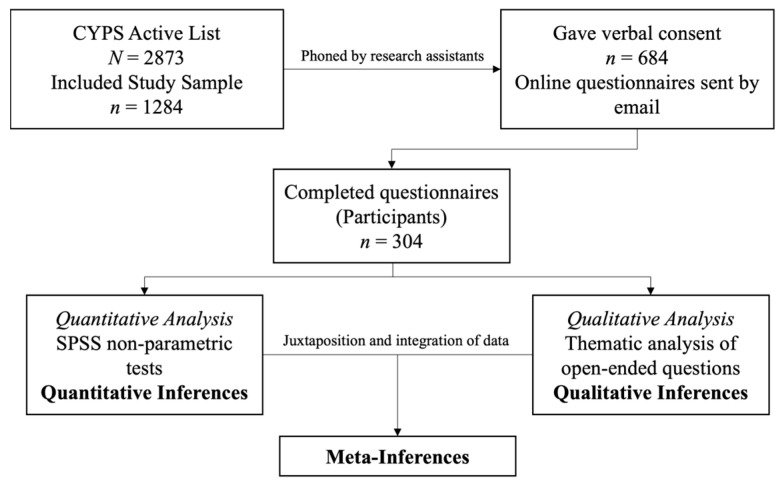
The study design.

Using the national CYPS patient database, 1284 YP met the inclusion criteria (Figure 1). Eight research assistants (junior doctors and psychiatry specialist trainee doctors) phoned the parents/caregivers of all the YP to offer information about the research, obtain verbal consent, ask for their preferred email address to receive the online questionnaire, and answer any other relevant queries. Being medical professionals, the research assistants had experience in communication skills with patients and relatives. Nevertheless, they were extensively briefed about their role over a series of sessions prior to contacting the participants. During the process, participants were assured that their responses (or lack of) would have no impact on the quality of their healthcare provision or therapeutic alliance.

In total, 684 (53.27%) of the contacted participants gave their verbal consent to receive the online questionnaire, which included an embedded written consent form within it. Of these, 304 (44.44%) participants returned the fully completed questionnaire. Therefore, 23.68% of the total chosen population (*n* = 1284) completed the questionnaire following three reminders, consistent with typical response rates for online questionnaires [29]. Based on the total available population (*N* = 1284) and a margin of error of 5%, the sample size calculation resulted that a minimum sample of 304 participants was required for this study, thus supporting our sample size (*n* = 304). The following equation was used [30]:

$$n=\frac{N}{1+N{\left(e\right)}^{2}}$$

### 2.4. Data Analysis

The quantitative variables analyzed were age (years), gender (male/female), locality districts (Table 1) [31], school educational level (secondary school—grade 7–9, secondary school—grade 10–11, post-secondary school), MD, engagement in sport, and GLTEQ score.

Quantitative data were analyzed using Statistical Package for Social Sciences (version 29.0.1.0) (SPSS; IBM Corp. 2023, NY, USA). The Kolmogorov–Smirnov test indicated that the two continuous variables in this study did not follow a normal distribution: age, D(304) = 0.17, *p* < 0.001; and GLTEQ score, D(304) = 0.14, *p* < 0.001. Furthermore, the other three variables were categorical (gender, locality, school year). Therefore, non-parametric statistical tests were used, namely the Mann–Whitney *U* test, Kruskal–Wallis test, Spearman correlation test and ordinal regression analysis for non-parametric continuous variables, and the chi-squared test and the Fisher’s exact test for categorical variables. The Fisher’s exact test was used (instead of the chi-squared test) on one occasion to analyze the association between locality and MD, as more than 20% of the cells had expected frequencies of less than 5 [32]. Eta squared (*η*^2^), Spearman’s rho (*r*_s_), and phi (*Φ*) were used as effect sizes. Effect sizes of *η*^2^ = 0.01, 0.06, and 0.14 indicated small, medium, and large effect sizes, respectively; *r*_s_ = 0.1, 0.3, and 0.5 indicated small, medium, and large effect sizes, respectively; *Φ* = 0.05, 0.10, 0.15, and 0.25 indicated weak, moderate, strong, and very strong effect sizes, respectively [33].

Reflexive thematic analysis (RTA) [34] was used to analyze the supplementary qualitative data from the open-ended questions; comparable to previous research [35]. The first author read the answers to the open-ended questions in detail, immersing himself in the data to gain a deeper understanding, a process called familiarization. The data were then assessed for significant patterns and labeled accordingly, assigning codes (in the form of keywords or short sentences). Codes were then grouped into subthemes and further grouped into themes. The themes were carefully reviewed by the other researchers to ensure that they accurately represented the data. The themes were subsequently refined and defined, enabling the researchers to construct a coherent narrative that captured their essence. All the researchers upheld theoretical flexibility and reflexivity, ensuring that they were not merely passive observers in the theme development process, but were actively engaged in discussions and critical thinking, drawing on their field experience. This engagement helped ground the researchers’ interpretations in the data while remaining aware of their biases and preconceptions. The overall analysis was inductive, as the coding process did not adhere to any pre-existing theories [36]. The qualitative data were juxtaposed and integrated with the quantitative data using a side-by-side joint display as part of a quantitatively driven mixed methods design, generating meta-inferences. No software tools were used to aid in the qualitative analysis.

### 2.5. Rigor and Ethics

To enhance the rigor of the qualitative analysis of this research, the researchers aligned with the relativist approach of judging qualitative research [37]. This meant that the analysis distanced itself from a criteriological approach, utilizing open-ended concepts that are not fixed or predetermined. Reflections were carried out within the research team, with researchers acting as “critical friends” exploring alternative interpretations.

Ethical approval was obtained from the Health Ethics Committee on the 22 December 2023 (HEC20/23—Ministry for Health and Active Ageing, Malta). Research governance approval was obtained from the Chairman of the Department of Psychiatry of the Malta Mental Health Services. The study participants were given detailed information prior to participating in the research and were given the opportunity to ask any questions they might have. Written informed consent to participate was obtained before starting the questionnaire. The designated link directed the participant to a written consent form where the participant was encouraged to read the form carefully. Upon ticking “I agree to participate”, the software directed the participant to the first page of the questionnaire. All data were stored anonymously in a password-protected spreadsheet in an encrypted computer.

## 3. Results

### 3.1. Quantitative Results

#### 3.1.1. General Results

The participant age ranged from 12 to 18 years (*M* = 14.54; *SD* = 1.67) and included 191 males and 113 females. The Mann–Whitney *U* test indicated no significant difference in age between male and female participants in the sample, *U* = 10,739.50, *Z* = −0.07, *p* = 0.94. From an educational level standpoint, 131 participants were in Grade 7 to Grade 9, 94 participants were in Grade 10 to Grade 11, and 79 participants were in post-secondary education. chi-squared tests revealed no significant difference in the relation between gender and educational level, *X*^2^(2, *N* = 304) = 0.249, *p* = 0.88, and between locality and educational level, *X*^2^(8, *N* = 304) = 14.38, *p* = 0.07.

Participants resided at the Malta Southern Harbor district (*n* = 74), Northern Harbor district (*n* = 81), Southern Eastern district (*n* = 55), Western district (*n* = 38) and Northern district (*n* = 56); no participants resided in the Gozo and Comino district. The Kruskal–Wallis test revealed a significant difference in participants’ age across the five districts, *H*(4) = 10.69, *p* = 0.03, *η*^2^ = 0.02. The district with the highest mean rank (MR) was the Western district (MR = 189.74), while the district with the lowest was the Northern district (MR = 133.29). Consequently, post hoc comparisons using Dunn’s method with a Bonferroni correction for multiple tests revealed that the age MR of participants from the Western district was significantly higher than the Northern district, *p* = 0.02. The chi-squared test revealed no significant association between gender and locality, *X*^2^(4, *N* = 304) = 1.24, *p* = 0.87.

Of the total sample (*n* = 304), participants reported being followed up at the CYPS for various MDs (Table 2). The Kruskal–Wallis test revealed a significant difference in participants’ age across the eight disorder groups, *H*(7) = 22.75, *p* = 0.002, *η*^2^ = 0.05. The disorder group with the highest age MR was obsessive–compulsive or related disorders (MR = 322.96) while the disorder group with the lowest was disruptive behavior or dissocial disorders (MR = 208.03). Post hoc comparisons using Dunn’s method with a Bonferroni correction for multiple tests revealed that the age MR for the mood disorders group (MR = 317.03) was significantly higher than the age MR for the obsessive–compulsive or related disorders (MR = 208.03; *p* = 0.01) and the attention deficit hyperactivity disorder (ADHD) group (237.85; *p* = 0.03). The Fisher’s exact test (*p* = 0.38) did not indicate a significant association between locality and disorder group.

#### 3.1.2. Physical Activity

##### Physical Activity Across Demographic Variables and Sport Practice

Of the total sample, 174 (57.24%) participants practiced PA/sport, while 130 (42.76%) did not. Despite this, the GLTEQ scores of all participants (*n* = 304) were acknowledged for analysis, given that some participants who claimed that they did not do any PA/sport might have still carried out some form of mild PA and scored in the GLTEQ. The largest proportion of these practiced football (25.86%, *n* = 45), athletics (15.52%, *n* = 27), swimming (9.77%, *n* = 17), gym/bodybuilding (9.20%, *n* = 16), dancing (6.90%, *n* = 12), and basketball (6.32%, *n* = 11). The mean participant GLTEQ score was 33.29 (*SD* = 30.98). The Mann–Whitney *U* test indicated that participants who practiced a sport (MR = 190.41) overall carried out more PA than those who did not practice a sport (MR = 101.76), *U* = 4713.50, *Z* = −8.72, *p* < 0.001, with a large effect size (*η*^2^ = 0.25), emphasizing the role of a formal sporting discipline to bolster PA levels. Furthermore, the chi-squared test revealed that more males practiced sporting disciplines when compared to females, *X*^2^(1, *N* = 304) = 9.25, *p* = 0.002, with a strong effect size (*Φ* = 0.17). The Mann–Whitney *U* test also revealed that males (MR = 164.98) were significantly more physically active than females (MR = 131.41), *U* = 8408.00, *Z* = −3.23, *p* = 0.001, with a small effect size (*η*^2^ = 0.03). Ordinal regression analysis revealed that males were 1.98 times more likely to carry out PA than females (OR = 1.98; 95% CI, 1.31 to 2.98, Wald χ^2^[1] = 10.64, *p* = 0.001).

Spearman’s correlation analysis revealed no significant correlation between PA levels and age, *r*_s_(302) = −0.01, *p* = 0.88. Furthermore, the Mann–Whitney *U* test revealed no significant difference in age between those practicing sporting disciplines (MR = 157.03) and those who do not (MR = 146.44), *U* = 10,522.50, *Z* = −1.06, *p* = 0.29. The Kruskal–Wallis test resulted in no significant differences in PA levels across six locality districts, *H*(4) = 3.30, *p* = 0.51, and between different years of school education, *H*(2) = 0.90, *p* = 0.64. The chi-squared test also revealed no significant association between sport practice and locality, *X*^2^(4, *N* = 304) = 2.63, *p* = 0.62, and between sport practice and school educational level, *X*^2^(2, *N* = 304) = 4.72, *p* = 0.09.

##### Physical Activity Across Disorder Groups

The Kruskal–Wallis test revealed a significant difference in PA levels across the eight disorder groups, *H*(7) = 16.59, *p* = 0.02, *η*^2^ = 0.02. The disorder group with the highest PA MR within this significant model was PDs and related traits while that with the lowest was obsessive–compulsive or related disorders (Table 3). Post hoc comparisons using Dunn’s method with a Bonferroni correction for multiple tests did not generate any significant results. However, this might have been due to the presence of numerous factors (eight disorder groups) and multiple pairwise comparisons, leading to a very low corrected alpha (*α*) level (Bonferroni correction = *α*/*n* = 0.05/8 = 0.006) to minimize the presence of Type 1 error [38]. Confirming this, individual Mann–Whitney *U* tests revealed several significant differences (Table 4).

### 3.2. Qualitative Results

The qualitative analysis generated nine subthemes which were further grouped into three main overarching themes (Table 5).

#### 3.2.1. Theme 1: Sports and Mental Health—A Mutual Alliance

##### Subtheme 1A: “An Active Body Contributes to a Healthy Lifestyle and Healthy Mind”

Participants generally highlighted the positive effects of PA, emphasizing its contributions to physical health, mental wellbeing, and overall quality of life, while showcasing the significant role sports play in personal development. The overwhelming majority of participants (94.08%, *n* = 286) believed that having good mental health contributes positively to sports performance and enjoyment, while an even higher proportion (96.71%, *n* = 294) believed that engaging in sports benefits YPs mental health:

*Good mental health is enhanced through sports and vice versa. A person who practices sports feels energized and more motivated to do other day to day activities. Being mentally resilient gets an athlete to perform much better than one who isn’t. People who do sports are able to cope better with stress and anxiety in their everyday life.*

(P41)

Doing a sport was regarded as an important step to “keep physically fit” (P3), but also had other benefits, such as feeling “energized… concentrated and focused” (P39). Several participants underscored the importance of sports in maintaining a level of “personal equilibrium and self-discipline” (P10), which “helps young people stay out of a rut and always have something to work towards” (P26). Several participants shared their perspectives on sports in relation to their experiences with mental disorders. Some expressed caution regarding their engagement in sports: “When it comes to eating disorders, it’s not extremely helpful as it becomes a form of compensation for eating” (P28). Conversely, others reported that participating in sports positively impacted their symptomatology:

*As we all know sports is beneficial to everyone, physically and mentally. I believe it is even more important for kids on the autism spectrum as it is a great way to encourage them to socialize with others, makes them gain confidence in themselves and gives them the opportunity to discover the world around them other than being lost watching the tablet all day long.*

(P101)

##### Subtheme 1B: “Less Time on Technology, More Time to Socialize”

Participants explained that engagement in sports serves as a valuable alternative for YP, effectively reducing the amount of time spent on technological devices. They noted that sports facilitate social interaction, providing opportunities for YP to connect in a dynamic environment. By encouraging teamwork and communication, sport plays a pivotal role in enhancing the social development of YP, ultimately contributing to a more balanced lifestyle that prioritizes both physical and social wellbeing:

*I feel that physical activity helps them feel better… happier. It helps keep them away from electronic devices, it may also help them to understand the importance of healthy eating. It will help them socially interact with other young people.*

(P190)

Team sports were reported to help participants “interact better socially and show better social behaviors” (P41) and consequently lead to “more successful social outcomes in life, such as more confidence in job interviews” (P41). Utilizing the sport as a prosocial experience may serve as a “boost to find friends” (P109), preventing “social isolation and loneliness” (P106), stemming from participants “using their tablet and spending hours on end just doing nothing” (P101):

*What really affects children with mental disorders is the sense of not feeling welcomed in a group. In team sports, they’d get used to the notion that you’re living for others, that you’re accepted in society. That you’re good like others. That you’re capable.*

(P283)

##### Subtheme 1C: “Good Mental Health Impacts Performance and Resilience”

Participants reported that having good mental health “plays a crucial role” (P46) within the overall sports performance, and contributes to improved resilience, and motivation, ultimately leading to better performance outcomes and a more enjoyable sporting experience: “Being mentally resilient gets an athlete to perform much better than one who isn’t.” (P41). Furthermore, some participants also mentioned the role of mental health during period of athletic injury: “It is also important for injury recovery. Psychological support can help athletes cope with the frustration and anxiety of being injured.” (P106).

Some participants spoke about the negative impact that herd mentality might have on athletes. They reported that “some sporting cultures put too much pressure on athletes to perform better” (P96). This invariably reduces the athletes’ ability to “enjoy themselves in a healthy environment” (P59), potentially leading to burnout and anxiety.

#### 3.2.2. Theme 2: Barriers to Sport Engagement

##### Subtheme 2A: “Lack of Support and Awareness”

This subtheme emerged prominently in the study, with numerous participants highlighting their experiences of marginalization in sports related to their mental disorders. They described varying degrees of exclusion, demonstrating how their mental health challenges impacted their ability to engage fully in sporting activities and their overall sense of belonging:

*Within the school, I do not think that my son is being given the opportunity to participate with his peers in sports activities because he is not encouraged to participate. If it is a team sport, he would be considered as a liability to the team.*

(P210)

In keeping with this, participants reported a “lack of appropriate facilities, including a lack of assistance to take their medication” (P248). The authors perceived a sense of helplessness in the participants’ responses, possibly stemming from multiple attempts to find the appropriate sports facility:

*Since he is on the autism spectrum, starting a sport is not that easy as there are rules to follow and they are not tailormade, apart from the fact that the individual might show no interest at all… Unlike neurotypicals, it might be hard to grasp.*

(P225)

##### Subtheme 2B: “Lack of Time”

Many participants expressed that lengthy hours spent on technology consumed time that could have been dedicated to sports, limiting their opportunities for engagement in healthier pursuits: “Online gaming and social media is becoming the biggest competition to physical exercise” (P49). There was a consensual belief towards sports becoming an integral part of the school syllabus: “Sports should be given equal importance to academic subjects from a young age and integrated in the school syllabus (P29). Participants raised concerns about the “long and strenuous school hours, and lots of assignments” (P14), which left minimal time for sports participation:

*Our education system is way behind… Unfortunately, our education system promotes or even pushes youths to spend endless hours in a classroom, then home to do homework and study for continuous assessments. These hours result in much more than what an adult spends on a full-time job and is extremely unhealthy.*

(P10)

##### Subtheme 2C: “Expensive and Not in the Vicinity”

Several participants explained that engaging in sports is often a high-cost financial burden, limiting access to various sports activities. Reflecting on this, the authors are also cognizant that a large proportion of patients attending a national mental health service originate from low social class backgrounds with limited financial resources [39]: “Not everyone can afford to pay more for a sport/weekly workout when you’re paying for therapies and help with schoolwork” (P225).

Participants noted that geographical distance posed another barrier to sports participation. Furthermore, the expanding urbanization of society has been identified as a challenge and risk to YP who are trying to engage in outdoor sports: “Sometimes, it is not safe to jog outside due to traffic, so I prefer it for him to use the treadmill for the time being” (P41). Reflecting on this, we can link heavily urbanized societies to a greater occurrence of mental health symptoms, supported by a recent umbrella review and meta-analyses [40], and as mentioned by participants in our study: “He always says that he would take up running if the roads were safer and there was more greenery around… He feels “trapped” here” (P39).

#### 3.2.3. Theme 3: National Sport Mental Health Service

##### Subtheme 3A: “Integrating Physical Activity with Mental Health Support”

Tellingly, 97.70% (*n* = 297) of participants felt that a national sport mental health service for YP would be beneficial. Many participants felt that a service that “links the sports side together with the mental health issues would be really beneficial” (P22). Therefore, this service was perceived as an opportunity for YP with mental disorders to engage in sports and alleviate their mental health symptoms. Furthermore, having such a service was also regarded to be a cost-effective way of reducing mental health stigma:

*A sport and exercise mental health clinic within the NHS would provide a holistic approach to care, leveraging physical activity to enhance mental well-being, prevent mental health issues, and accelerate recovery. It could reduce stigma, be cost-effective, and contribute to research on effective treatments.*

(P33)

Participants felt that “encouraging exercise would alleviate the bottleneck to access mental health services” (P69). Such a service (using exercise prescriptions) would also function to prepare YP with MDs “to attend actual sports within organizations” (P99) and help them engage in sport programs based on their strengths:

*A clinic could suggest suitable sports programs for youths with specific mental health challenges. Such a clinic could provide more structured programs for these young people and set them in the path of recovery and/or a better quality of life.*

(P41)

##### Subtheme 3B: “Educating the Educators and Parents”

Participants felt that a national sport mental health service would serve to educate other professionals and individuals within the athlete’s ecological systems, such as family members and coaching staff. This may manifest in various forms, such as the presence of a mental health professional with the athlete and conducting educational talks, reflecting the service’s multimodal approach:

*There is a sport psychologist going around with them during training three days a week in case they need. Every month, they have an hour talk divided in two sessions. It helps them a lot and parents see the difference in their behavior towards sports. She also does a half an hour talk to us parents and to the coaches every month.*

(P16)

##### Subthemes 3C: “The Athlete Needs to Be Capable and Strong”

Traditionally, a sports mental health service is a clinic for athletes with mental health symptoms or who require psychological intervention. Participants believed that this specialized service would encourage athletes to be more open about their mental health symptoms: “A sports mental health clinic will surely help athletes see the services differently” (P215). Participants also felt that such a service “can offer a tailored exercise program designed to meet individual needs based on the athlete’s physical and mental health conditions” (P106).

Recognizing athletes as a distinct mental health population, participants highlighted the need for an effective therapeutic service that “could make a huge positive impact on the individual athlete” (P242). This could potentially alleviate some challenges associated with the athlete’s performance:

*An athlete can end up in several situations such as bullying and abuse. The athlete needs to be capable and strong, to not give up when things are going badly. Likewise, when things are doing well, it’s important for the athlete to remain humble and not lose the sense of sportsmanship.*

(P255)

### 3.3. Integration of Data

The supplementary qualitative results were integrated into the main qualitative component to generate meta-inferences (Table 6).

## 4. Discussion

The results from this study showed that participants who practiced a sport were significantly more physically active than those who did not. Males were significantly more physically active than females (1.98 times more likely) and carried out significantly more sports than females. A significant difference in PA levels was observed across the eight disorder groups; PDs and related traits had the highest MR, while obsessive–compulsive or related disorders had the lowest MR. No significant association was observed in PA levels and sport practice between different ages. Furthermore, no differences in PA levels/sport practice were observed across locality districts and school educational level. Supplementary qualitative data suggested that PA helps with both physical and psychological wellbeing, encourages social interaction, and has a positive link with mental health. Qualitative data identified barriers to carrying out sports/PA such as a lack of awareness, lack of time, and costly facilities. Results also highlighted the dual role of a national sport mental health clinic, including the psychoeducational and exercise prescription role on one hand, and the athlete mental health on the other.

The above results may be interpreted within the framework of behavior change models. In our study sample, male patients exhibited significantly higher levels of PA compared to females. Although similar findings have been previously documented [10], it is noteworthy to observe this gender association within the CAMH population. Possible reasons for this gender disparity in PA might be the different perceived locus of control. Previous research has indicated that females are more likely to engage in PA driven by extrinsic motivation, such as weight loss, whereas males are more likely to be motivated by intrinsic factors, such as the enjoyment of sport [41,42]. This aligns with the self-determination theory [43] which posits that intrinsic motivation (stemming from internal desires) serves as a stronger motivational force for change than extrinsic motivation (influenced by external incentives).

The athletes from our study were significantly more physically active than the non-athletes. While this may seem to be an obvious finding, one may appreciate that a sizeable proportion of YP carrying out PA do not do so in the context of an organized sport [44]. This may include house chores, traveling and walking, among others. However, being part of an organized sport has been linked with increased PA levels [45], possibly as it provides a sense of meaning, identity, and belonging in both healthy individuals and mental health patients [46]. Furthermore, team sport or individual sport within clubs fosters a stronger shared social identity, harboring a greater sense of “us” and togetherness [47], possibly improving their level of commitment [48]. Considering the transtheoretical model of change [49], most established athletes would already be in the “maintenance” phase (doing sports consistently for more than six months) while non-athletes might still be in the “pre-contemplation” or “contemplation” phases, including the ambivalence towards PA therein.

Patients with PDs and related traits significantly reported the highest levels of PA while those with ODD/CD were the least physically active. Dialectical behavioral therapy is one of the standardized therapeutic modalities used in patients with PDs and related traits to enhance positive coping [50]. Consequently, this might convince these YP to use PA as a coping strategy to instill positive emotions [51]. Conversely, the lack of structured routine associated with ODD/CD patients [52] may discourage them from becoming physically active.

Both patients and their caregivers identified multiple barriers related to sport and PA, most of which were environmental barriers, such as a lack of awareness about mental health, issues with accessibility, and a lack of trained staff or appropriate facilities. As a construct within social cognitive theory [53], reciprocal determinism describes the dynamic triangulation between personal factors, the environment, and behavior change, which all interact with one another. Using this theory, perceived environmental barriers might disincentivize YP from carrying out PA (behavior change). Therefore, in addition to internal motivators, environmental and social encouragement are crucial to improve the overall engagement in PA and sports. Most participants believed that a national sport mental health clinic will be beneficial to safeguard the mental health of athletes and to promote PA in the overall care plan of YP with MDs. PA can prevent MDs [54], and has shown medium effects on depression, anxiety, and ADHD [55,56]. A randomized controlled trial [57] consisting of eighty-six college students with depressive symptoms found that almost three-quarters (72.09%) of participants improved to “normal” mood at the 12-week mark following the implementation of exercise prescriptions. This affinity towards exercise prescriptions might be the result of the person-centered approach in mental health, which has been becoming increasingly prevalent over the past years [58].

So, why are the results obtained from this study relevant to modern research and current service development? Firstly, the paucity of sport/PA research in young mental health populations adds a novel dimension to findings presented in the current literature. Secondly, due to already overstretched budget systems and the consequent need for efficient healthcare budget allocations [59], such results may serve as clinical guidance by prioritizing the need for exercise prescription and athlete identification within socio-demographic groups. For instance, given that results from this study report low PA levels in patients with disruptive behavior or dissocial disorders, clinicians may identify a special need to prescribe PA as a behavioral coping strategy in this sector. Thirdly, these results focus on barriers related to sports/PA in mental health patients and the lack of specialized services for athletes experiencing mental health symptoms. As highlighted by the Lancet Global Health Commission for High Quality Health Systems, system level changes such as service delivery models are required to drive systemic change [60]. Such results may generate population demand for health system quality, potentially motivating political leaders (who tend to be vote-driven) to deliver and invest in a multimodal national sport mental health service [61]. Lastly, such research highlighting the need for accessible sport mental health clinics within the public service supports the notion that such services should embrace cultural diversity and social justice, shaping a more inclusive future in sport psychology. This notion has also been supported by the International Society of Sport Psychology’s position on cultural praxis in sport psychology [62]. A national sport mental health clinic will help mitigate the identified barriers in this study by providing good and free (within a tax-based health system) access to care in a timely manner, by maintaining the same level of professionalism and confidentiality expected in a national mental health service, and by streamlining trained professionals to offer a more specialized service when compared to the general population.

### Strengths and Limitations

This study was subject to certain limitations. Response bias might have been introduced due to the self-reported measure used [63]. Potentially, certain responses about PA levels might have been purposefully hyperbolized since participants were aware that this was a study about PA and sports. However, parents/caregivers were encouraged to complete the questionnaire together with the child to clarify answers. The Hawthorne Effect might have also generated response bias as participants might have changed their responses due to the awareness of being studied by a healthcare professional [64]. To minimize this bias, submitted questionnaires were anonymous and all participants were informed that their results will have no impact on their healthcare provision. Since the quantitative arm of this study was cross-sectional, this limited the ability to generate causal inferences [65]. Nevertheless, the qualitative results from this study helped give more insight into potential causes. Furthermore, the questionnaire was not tested for reliability and validity. However, the GLTEQ within the questionnaire has been tested by the developers of the tool [25], and the other section of the questionnaire relates to descriptive and qualitative data. The primary strengths of this study are its innovative approach and the use of a mixed methodology. To the authors’ knowledge, this is the first mixed method study analyzing PA levels in a national CAMH population. The mixed methods approach capitalized on the strengths of both methods, compensating for each other’s weaknesses [66]. The quantitative component helped generalize the findings, while the qualitative component provided a deeper understanding of the patient’s and caregiver’s experience [67].

## 5. Conclusions

The male gender, athletes, and patients with PDs and related traits were associated with the highest PA levels. Participants reported several barriers they face when planning to engage in PA, including a lack of awareness regarding mental health, insufficient access to suitable sporting facilities, the distance of facilities from their homes, and financial constraints, among others. A significant majority of participants agreed with the development of a service aimed at alleviating these barriers, while providing a more specialized and effective resource for athletes and individuals seeking to incorporate exercise into their therapeutic regimen.

Future recommendations include the planning of interventional and longitudinal studies to assess causality, especially with the inclusion of randomized-controlled trials. Another recommendation is the introduction of training sessions for healthcare professionals to highlight the importance of PA as an integral part of one’s therapeutic experience. The results of this study will serve to inform the development of the first documented sport mental health clinic for YP.

## Figures and Tables

**Table 1:** Maltese localities and population by districts.

District	Population	Localities
Southern Harbor	87,438	Birgu, Bormla, Fgura, Floriana, Kalkara, Luqa, Marsa, Paola, Santa Lucija, Senglea, Tarxien, Valletta, Xghajra, Zabbar
Northern Harbor	168,636	Birkirkara, Gzira, Hamrun, Msida, Pembroke, Pieta’, Qormi, St. Julians, San Gwann, Santa Venera, Sliema, Swieqi, Ta’ Xbiex
Southern Eastern	79,498	Birzebbuga, Ghaxaq, Gudja, Kirkop, Marsascala, Mqabba, Qrendi, Safi, Zejtun, Zurrieq
Western	66,993	Attard, Balzan, Dingli, Iklin, Lija, Mdina, Mtarfa, Rabat, Siggiewi, Zebbug
Northern	99,295	Gharghur, Mellieha, Mgarr, Naxxart, St. Paul’s Bay
Gozo and Comino	40,191	Fontana, Ghajnsielem, Gharb, Ghasri, Kercem, Munxar, Nadur, Qala, San Lawrenz, Sannat, Xaghra, Xewkija, Rabat, Zebbug
Total	542,051	

**Table 2:** Distribution of disorder groups among study sample.

Disorder Group	*n*	%
Attention deficit hyperactivity disorder (ADHD)	170	55.92%
Anxiety or fear-related disorders (e.g., generalized anxiety disorder, panic disorder, social anxiety disorder)	130	42.76%
Autism spectrum disorder (ASD)	84	27.63%
Mood disorders (e.g., depressive disorders, bipolar disorders)	47	15.46%
Disruptive behavior or dissocial disorders (e.g., oppositional defiant disorder, conduct dissocial disorder)	40	13.16%
Personality disorders (PDs) and related traits (e.g., borderline pattern)	17	5.6%
Obsessive–compulsive or related disorders	13	4.28%
Disorders of intellectual development (ID)	12	3.95%

**Table 3:** GLTEQ mean ranks (Kruskal–Wallis Test) analyzing PA levels across disorder groups.

Disorder Group	Mean Rank
Personality disorders and related traits	350.12
Attention deficit hyperactivity disorder	281.33
Mood disorders	244.82
Anxiety or fear-related disorders	243.83
Autism spectrum disorder	239.20
Disorders of intellectual development	231.13
Obsessive–compulsive or related disorders	228.69
Disruptive behavior or dissocial disorders	225.50

**Table 4:** PA levels between disorder groups (individual Mann–Whitney *U* tests showing significant differences).

Disorder Group	MR	*p*	*η* ^2^	Result
ADHD	134.84	0.02	0.02	Patients with ADHD more physically active than patients with ASD
ASD	112.64			
ADHD	109.96	0.03	0.02	Patients with ADHD more physically active than patients with ODD/CD
Disruptive behavior/dissocial disorders	86.53			
PD and related traits	38.26	0.01	0.14	Patients with PD and related traits more physically active than patients with ODD/CD
Disrupt behavior/dissocial disorders	25.06			
PD and related traits	17.71	0.04	0.15	Patients with PD and related traits more physically active than patients with ID
ID	11.17			
PD and related traits	70.32	0.003	0.09	Patients with PD and related traits more physically active than patients with ASD
ASD	47.09			
ADHD	160.04	0.03	0.02	Patients with ADHD more physically active than patients with anxiety disorders
Anxiety and fear-related disorders	138.03			
PD and related traits	119.29	0.04	0.02	Patients with PD and related traits more physically active than patients with ADHD
ADHD	91.47			
PD and related traits	99.71	0.01	0.05	Patients with PD and related traits more physically active than patients with anxiety disorders
Anxiety and fear-related disorders	70.64			
PD and related traits	40.74	0.03	0.07	Patients with PD and related traits more physically active than patients with depressive disorders
Mood disorders	29.52			

**Table 5:** The themes and subthemes generated from the qualitative analysis of open-ended questions.

Theme 1: Sports and Mental Health—A Mutual Alliance	Theme 2: Barriers to Sport Engagement	Theme 3: National Sport Mental Health Clinic
Subtheme 1A: “An active body contributes to a healthy lifestyle and healthy mind”	Subtheme 2A: “Lack of support and awareness”	Subtheme 3A: “Integrating physical activity with mental health support”
Subtheme 1B: “Less time on technology, more time to socialize”	Subtheme 2B: “Lack of time”	Subtheme 3B: Educating the educators and parents
Subtheme 1C: “Good mental health impacts performance and resilience”	Subtheme 2C: “Expensive and not in the vicinity”	Subthemes 3C: “The athlete needs to be capable and strong”

**Table 6:** A table representing the integration of the quantitative and qualitative data.

Research Question	Quantitative Inferences	Qualitative Inferences	Meta-Inferences
Do PA levels differ across socio-demographic groups of YP with MDs?	(1) Significantly higher PA levels in those doing a sport (2) Significantly higher PA/sport levels in males compared to females (3) PA levels varied significantly among MDs (highest in PDs/related traits, lowest in disruptive behavior/dissocial disorders) (4) Age varied significantly among MDs (obsessive–compulsive/related disorders being the oldest group, disruptive behavior/dissocial disorders being the youngest.	(1A) PA improves physical and psychological wellbeing. (1B) Sports help YP reduce screentime and socialize more. (1C) Good mental health boosts athletic performance. (2A) Lack of support and mental health awareness in athletes. (2B) Lack of time to practice sports or PA. (2C) Expensive and distant sport facilities. (3A) Using PA prescriptions for YP with MDs. (3B) Important to educate teachers/parents. (3C) Athletes’ mental health must be taken seriously	Males and athletes did more PA than others. Sport clubs probably bolstered PA levels. YP with disruptive behavior or dissocial disorders (youngest cohort) had the lowest PA levels, potentially due to behavioral challenges and young age.
What are the perceived barriers of sports and PA levels in YP with MDs?			Barriers were identified (lack of mental health awareness, lack of time, inappropriate sporting facilities in the area, and costly programs).
What are the perceptions of YP with MDs, and their caregivers, about the development of a national sport mental health clinic?			Participants encouraged the development of a national sport mental health clinic, both for athlete mental health and to integrate PA in patient care plans.

## Data Availability

The data presented in this study are available on request from the corresponding author anonymously due to ethical reasons.

## Abbreviations

The following abbreviations are used in this manuscript:

ADHD	Attention deficit hyperactivity disorder
ASD	Autism spectrum disorder
CAMH	Child and adolescent mental health
CYPS	Child and Young People Services
GLTEQ	Godin Leisure-Time Exercise Questionnaire
ID	Intellectual disability
MD	Mental disorder
MR	Mean rank
NSO	National Statistics Office
OR	Odds Ratio
PA	Physical activity
PD	Personality disorder
RTA	Reflexive thematic analysis
SPSS	Statistical Package for the Social Sciences
WHO	World Health Organization
YP	Young people
